# Ante-Natal Pathology

**Published:** 1911-04-15

**Authors:** N. S. Hoff


### ANTE-NATAL PATHOLOGY.

BY N. S. HOFF, D.D.S.

---

**Embryonal Pathology.** Pathology of the embryonic period of intra-uterine life must have reference to diseases or malformations of the primary tissues and organs in the earliest history of the foetus. Accidental or morbid agencies acting upon the foetal organism during its formative period would naturally produce structural anomalies and deformities, or seriously retard development, whilst infectious diseases might result in death and abortion.

As the dental organs do not begin their development until the close of the embryonic period their anomalies can not be properly referred back beyond the so-called foetal period, which begins about six weeks after impregnation. Therefore we must attribute to embryonic disturbance only such aberrations as can be classified under monstrosities rather than as dental anomalies. The embryonic period may then be known as the teratogenic rather than as the pathogenic period. We shall however, find that the foetal period may be as truly pathogenic as the post-natal period. We can then attribute to the embryonic period only such general conditions as would result in poorly formed tissues and organs; conditions which might influence dental development detrimentally to the extent of failing to supply the fundamental tissues for the development of adequate dental organs. We leave out of consideration the teratologic features of the problem of embryonal pathology because they have no particular connection with or practical bearing on the general subject which we are considering.

**Foetal Pathology.** The general subject of ante-natal pathology concerns itself with the many facts of foetal maldevelopment that have been noticed during the passing years and which have only recently been collected systematically to be studied with reference to preventive medicine and their special etiologic factors. Because many post-natal diseases can be traced back to ante-natal antecedents the etiologic significance of which are clearly established it is now possible to formulate scientific basic principles for ante-natal hygiene. In the past most of the work on this subject has been directed toward the betterment of the general health, but as no benefit can come to the general physical welfare that would not favorably react on the developmental processes for good teeth, even advice of a general nature will be valuable. We can not of course here give an adequate statement of all the probable applications of this fundamental work to dental prophylaxis but we shall call attention to some phases of it of more than casual interest.

While it is not fully established that there is always and of necessity ancestral transmission of specific taint or physical defects, it is very generally conceded that a degenerative tendency and a structural weakness is present as a strong predisposing factor in perpetuating disease especially when the principles of hygiene are ignored. Incidentally it is known that decided improvement in vital resistance can be secured by early prophylactic intervention. We have much evidence in malformed dental organs to convince us of accidental interference with normal function in the ante-natal development of the dental embryonic tissues, but we have not yet studied the tooth tissues with reference to the nature and cause of their structural weakness and consequent inability to resist disease invasions. We may therefore, be forced to adopt some of the teachings of heredity, such as Weismann's doctrine of the continuity of the germ plasm, and go back to the very inception of life for a basic principle in our prophylaxis. Historically we have small resources either in tradition or literature to furnish data of value on intra-uterine pathologic predispositions. The ancients considered the birth of child with a peculiar abnormal or monstrous development as an omen of peculiar significance, and these deformities have always been matters of interest and have given rise to much speculation as to their cause; more recently we have considered them as freaks of foetal development, and have probably underestimated their true pathologic significance. We have similarly neglected the study of pathologic and abnormal dental structures, because they appear even less conspicuous and spectacular. The study which Dr. Black has made of the enamel with reference to cavity preparation, and the experience which workers in modern prophylaxis methods are having with the cementum and peridental tissues, lead us to believe that we must have a better knowledge of the tooth tissues and their pathologic states. Much good work has been done by our dental histologists and we are fairly well acquainted with the normal histology of the teeth and their environment, and we also know something of the pathologic processes found in these tissues, but until we know more of the conditions which render possible these pathologic states we can not hope for successful prevention by any prophylactic treatment that we have been accustomed to prescribe. We must know how to prevent disease to which mothers are subjected from producing harmful affections in the foetus, because injurious impressions during the developmental period are irreparable. No other period in the life of an individual is so important from the standpoint of future health as the ante-natal period; and since it is known that diseases which affect the mother during this period are very likely to interrupt normal developmental processes, and may at times entirely suspend life, the problem of maintaining a well balanced equilibrium of health generally, and of preventing all forms of infectious diseases becomes a most important procedure.

We can not at present state exactly what impressions are made on the foetus by accidental or acquired diseases. Fortunately clinical findings show that mothers do not always transmit their physical infirmities to their children; traumatic injuries do not necessarily leave a mark on the child, nor are serious impressions always produced on the developing foetus by acute diseases, especially when not intense or prolonged, which may affect the pregnant mother. It is also a clinical fact that the same disease does not produce similar results at all ages. Many diseases that are easily endured by the child are attended with serious consequences in the adult or aged, and we find that many serious diseases of the mother produce no corresponding injury on the foetus, so far as can be determined by our present means of diagnosis. But while no apparent injury results during the intra-uterine period from the many maternal influences of a pathologic kind, the hypothesis may most reasonably be maintained that because of such maternal incidents typically normal intra-uterine development seldom obtains. May we not believe, for instance, that the prevalence of malocclusion in both temporary and permanent dentitions, is due to the many degenerating forces which have interfered with normal development of the tissues which construct the dental organs? If we can determine what maternal affections during the period of gestation are liable to produce harmful results in the development of good tooth tissues and organs, we shall have reached a practicable starting point of eliminating the essential factors which stand in the way of typically normal dentition. We shall at least be able to insist on more consistent and rigorous application of specific rules of prevention, or better a hygienic regimen. In order that we may direct attention to the maternal influence in producing pathologic conditions in the child we shall need to study specifically the conditions which have been held responsible for the malformations and diseases occuring in utero.

**Heredity.** The old theory was that all forms of malformation and disease in the child were transmitted by the parents in some direct but unknown manner. The Greeks made of it a proverb, "a bad crow lays a bad egg." Many writers still maintain that all forms of irregularity in development are transmitted due to direct transmission. We find writers who maintain that malocclusion of the teeth is due to the child inheriting one parent's type of teeth and the other parent's jaw bones. W. Pfaff has a record of 100 children belonging to 50 families presenting anomalies so similar to those of their parents or grand parents that it would seem that there was a large element of truth in the theory, especially when it appears that these anomalies reappear in the same form and location. This theory also finds much support in efforts made by animal breeders and botanists to exaggerate certain characteristics by emphasizing the law of hereditary selection. On the other hand we find that these principles of development are liable to be changed by environment and accidents. For instance, there seems to be no such thing as stability in the natural order, and where neglect or unnatural conditions predominate a process of degeneration becomes operative. While nature seems to establish a type, extraneous causes when active bring about a typical modification, sometimes to the extent of monstrosities.

The more recent thought of scientists seems to be that disease is not transmitted as such on any hereditary basis, and that no more than a predisposition to disease is thus inherited; this predisposition manifests itself in the tissues and organs as lowered vital resistance. It is also thought that many diseases supposed to have been transmitted hereditarily are due to direct pathologic infections. Various forms of neuritis, cellulitis, cancer, etc. are not now believed to be directly transmitted, but degenerate tissue forms a basis for subsequent development of these diseases when favoring conditions predominate. It is generally conceded that such diseases as measles, scarlet fever, small-pox, syphilis, erysipelas, typhoid fever and other eruptive fevers, which make a strong impression on the mother's vitality and extend over a long period during pregnancy undoubtedly seriously affect intra-uterine development, sometimes to the extent of devitalization, and it is generally suppossed that all such accidents during the latter part of the intra-uterine life make their impressions upon the enamel organs and may lead to serious pathologic results in the newly-born unless its early foetal development has been so strong as to enable it to pass a critical period uninfluenced by the maternal affection. The etiology of such diseases as rickets and scrofula is somewhat obscure and the maternal influence is difficult to explain. We can not say that these diseases are transmitted in the ordinary sense and yet they are certainly acquired congenitally. In all such affections we find the teeth defective when erupted and in many cases serious malformations. Owing to the utter silence of most of our text books and the barrenness of our current literature on this subject we are unable to present a conclusive statement as to the etiologic factors, or a rational suggestion as to specific treatment. Dr. Black in his Text Book on Operative Dentistry, Vol. 1, page 13, says he has no doubt that measles, whooping cough, scarlatina and hereditary syphilis are responsible for the pittings in childrens' permanent teeth. He also relates having observed several cases where he positively expected such a result only to be agreeably surprised to find the enamel perfectly formed when the teeth were erupted. If there is such indefiniteness as to these influences in post-natal life, how can we expect to trace such a cause back to some ante-natal influence, or suggest a preventive remedy? As we have noted above we must credit the vital resistance which carries a child through the many vicissitudes of childhood and robust youth to manhood, to a well nourished and unaffected embryonic and foetal existence. If this premise has facts enough of a clinical nature to substantiate its truth we are then prepared to deduce the opposite conclusion that post-natal development defects and diseases are in a large measure due to malnutrition and pathologic interruptions of foetal development. We are forced to conclude that there is such a thing as ante-natal pathology because abortions are so common, and only a comparatively small proportion of them are traceable to external or accidental causes. If disease affects the foetus to such a fatal extent in some cases it is reasonable to suppose that it may have been actively present in less serious degree during the ante-natal period. If this is true may not these affections account for the lapses to be found in later development of such tissues as compose the generative organs of the teeth? We must conclude that there is an ante-natal pathology, and if so we must find a remedy that is practicably applicable. Much research is yet to be made to determine the cause of those subtle tissue aberrations which produce the many varieties of oral and dental abnormalities.

Professor Warnekross of Berlin has promulgated a theory after long study and research that hare lip and cleft palate conditions are directly due to the presence of supernumary teeth, and he cites a large number of cases to substantiate his theory. Dr. C. J. Lyons of Michigan confirmed this theory by a number of cases that have come under his observation. It will be the next step to find out why we have supernumary teeth, and then we must discover a method of preventing their formation, or at least their tendency to cause mischief. A knowledge of the cause of dental anomalies and diseases is essential to prevention. We can hope for definite knowledge at present only through logical deductions; we must observe and report our findings for future deductions and conclusions.

Anomalies of the teeth and jaws may be congenital or acquired. If congenital the cause may center around some disturbance during the developmental period or be traced back to inherited conditions. Walkhoff maintains that the mixture of races is a particularly fruitful cause of such anomalies. This view is also maintained by a considerable number of other observers. The majority seem inclined to the view that topographical anomalies of the teeth are generally congenital and but rarely acquired, and still while there may be a congenital predisposition toward such anomalies, in many instances the determining factors are to be looked for among the environmental influences. Among those who have written on dental anomalies are Cartwright,

Coleman, Coles, Mummery, Kingsley, Carabelli, Talbot, Schroder, Sternfeld, and Walkhoff.

**Traumatism and Pregnancy.** It is commonly believed that traumatic injuries to pregnant women are very likely to produce some form of injury to the developing foetus This belief is largely traditional and has little support in clinical evidence or scientific logic or research. The supposed injuries extend all the way from mental impressions and psychical shocks to actual injuries, and all sorts of congenital abnormalities have been attributed to such incidents. Charpentier, says, "When a perfectly healthy woman is injured during pregnancy, no harm results, unless the injury is to the genital tract, or there is extensive hemorrhage, or the wound should become infected with erysipelas, or lymphangitis, etc." We may infer from this that such accidents should not be considered at all important in malformations of the teeth, since the teeth develop late in intra-uterine life, and in the midst of well protected tissues where they would be only slightly susceptible. How far they may be influenced through shocks to the nervous system remains to be demonstrated. But owing to the fact that tooth tissue calcification is such a slow process it is highly probable that no acute disease or injury will have serious influence on its normal process of development. It could only prove seriously detrimental in prolonged convalescense or infections which would contaminate the blood supply and so interfere with nutrition at a critical period. The idea that the extraction of a tooth for a pregnant woman may produce sufficient mental impression to interrrupt the process of calcification is too visionary to deserve serious consideration. It is exceedingly doubtful if there is a close enough correlation between the psychical and sensational functions to influence trophic function in the tooth follicle. It would seem highly improbable that traumatism of any kind which produces only a brief impairment of normal function could have any considerable influence in modifying enamel formation, especially during the intra-uterine period. What the effect on the embryonic dental structures might be is not apparent.

**Infectious Diseases of the Foetus.** It is a fairly well settled fact that the foetus may sustain attacks of diseases to which it is liable after birth. These diseases are mostly of the infectious type. A discussion of this subject appears in medical literature as early as 1837, in a book written by Graetzer, entitled, "Krankheiten des Foetus," and again in a book by Ballantyne, 1892, on "Diseases and Deformities of the Foetus." There is also considerable to be found in current medical literature on the subject. None of it, however, seems to have considered specifically the influences of intra-uterine diseases on the development of the teeth.

The foetus is protected from external infectious influences by its isolation and environment; in spite of which, it is in many cases attacked with the same diseases to which a the child is exposed after birth. Some of these prolonged intra-uterine diseases may, in part at least, account for certain dental and oral malformations. One class of these diseases would include the exanthemata, malaria, tuberculosis, etc. Another class the toxicological diseases, such as lead and phosphorus poisoning, mercury, morphine habit, intemperance, and other drug habits. A third class would include the so-called idiopathic diseases, such as diseases of the nutrient tracts, neuritis, etc. Small-pox in the foetus has been known since 1702 when Duttel cited several cases. In some of these cases the mother seems to have escaped the disease; it seems peculiar that the foetus should receive the infection and the mother at least express no outward symptoms of the disease. This would lead to doubt as to the validity of the statement, but some of the children were born with the indications of small-pox on their bodies. In some cases where pregnant women have suffered from small-pox children have been born with the eruption on their bodies. Charcot, in Compt. Rend., Societe de Biol. (1853, Vol. V, page 88), reports a case of foetal *variola* in which there were fifteen characteristic spots on the face and others on the mucous membrane of the mouth. We cite this to show that it is possible for the foetus not only to be subject to infectious diseases, but also to recover from the same before birth. Undoubtedly the possibility of such attacks account for many of the deformities which we recognize as being caused by impeded development, such as malocclusion, cleft palate and hare lip. The *Journal de Med. Chirurg et Pharm.*, (Vol. I, page 145,) reports a case of necrosis following an infectious disease during foetal life. Ballantyne reports twenty cases of *measles in utero.* The *Edinburgh Medical Journal*, 1893 page 13, reports a case of *scarlet fever* in utero, diagnosed at the birth of child, the mother having experienced the symptoms of scarlet fever during the seventh month of pregnancy. Few such cases are recorded, but probably many more exist that are either unrecognized or remain unreported. E. Bidone, in *Teratologia*,—1894, page 182,— relates a case of intra-uterine erysipelas in which the child died nineteen hours after birth, and the necropsy showed infection of the spleen, lungs, kidneys and heart. This disease has been recognized by other observers as occuring during intra-uterine life. Many other diseases have been reported as affecting foetal life either directly or reflexly from the affections of the mother. Such as relapsing fever, yellow fever and influenza, etc.

Medical literature is full of citations that if studied with regard to their influences on foetal development of the dental system would undoubtedly throw much light on dental anomalies and defective tooth structures. The important question with us is, how are we to meet this emergency and apply practical preventive measures? Dentists are seldom consulted on such matters, and physicians do not consider them of great importance. If we are to do anything radically corrective it would seem that the only way open to us is to begin a campaign of education. We must present this subject to our patients, who may be interested, in an intelligent manner, and with their help we shall be able to impress the medical practitioners with the importance of its bearing not only on good teeth, but on all other structures and organs of the body. At present we must agitate on a somewhat tentative basis, and in the meantime, observe and report all cases which may help to collect the necessary data for the establishment of a definite scheme of prophylactic hygiene.

